# The antibiotic furagin and its derivatives are isoform-selective human carbonic anhydrase inhibitors

**DOI:** 10.1080/14756366.2020.1752201

**Published:** 2020-04-16

**Authors:** Aleksandrs Pustenko, Alessio Nocentini, Paola Gratteri, Alessandro Bonardi, Igor Vozny, Raivis Žalubovskis, Claudiu T. Supuran

**Affiliations:** a Latvian Institute of Organic Synthesis, Riga, Latvia; b Institute of Technology of Organic Chemistry, Faculty of Materials Science and Applied Chemistry, Riga Technical University, Riga, Latvia; c Department of NEUROFARBA, Section of Pharmaceutical and Nutraceutical Sciences, University of Florence, Firenze, Italy; d Department of NEUROFARBA, Section of Pharmaceutical and Nutraceutical Sciences, Laboratory of Molecular Modeling Cheminformatics & QSAR, University of Florence, Firenze, Italy

**Keywords:** Carbonic anhydrase inhibitors, molecular dynamics, furagin, hydantoin, synthesis

## Abstract

The clinically used antibiotic Furagin and its derivatives possess inhibitory activity on human (h) carbonic anhydrases (CA, EC 4.2.1.1), some of which are highly expressed in various tissues and malignancies (hCA IX/XII). Furagin exhibited good hCA IX and XII inhibition with *K*_I_s of 260 and 57 nM, respectively. It does not inhibit off-target CA I and poorly inhibited CA II (*K*_I_ = 9.6 μM). Some synthesised Furagin derivatives with aminohydantoin moieties as zinc binding group exhibited weak inhibition of CA I/II, and good inhibition of CA IX/XII with *K*_I_s ranging from 350 to 7400 and 150 to 5600 nM, respectively. Docking and molecular dynamics simulations suggest that selectivity for the cancer-associated CA IX/XII over CA II is due to strong H-bond interactions in CA IX/XII, involving the tail orientated towards hydrophobic area of the active site. These results suggest a possible drug repurposing of Furagin as anti-cancer agent.

## 1. Introduction

Carbonic anhydrases (CAs, EC 4.2.1.1) are ubiquitous metalloenzymes, being encoded by at least eight different genetic families, which have been found in organisms all over the phylogenetic tree^1–10^. CAs catalyse a crucial physiologic reaction, where by hydratation of CO_2_ is formed a weak base (bicarbonate) and a strong acid (hydronium ions). These enzymes are involved in a multitude of physiologic processes, starting with pH regulation and ending with metabolism^1–3^^,^^7–10^^,^^11–22^.

CAs are also involved in various pathological processes and therefore are drug targets for decades, with their inhibitors having pharmacological applications in many fields^1–3^^,^^7–19^. The primary sulphonamides were discovered as CA inhibitors (CAIs) already in the 40 s, and most of the drugs that were launched in the next decades as diuretics, antiepileptics, or antiglaucoma agents targeting CAs belonged to this class of compounds^1–3^^,^^7–19^. Although highly potent as CAIs^1–3^, the sulphonamides generally non-selectively inhibit most α-CA isoforms present in humans and mammals in general^1–3^ as well as CAs from the other genetic families (β-, γ-, δ-, ζ-, η-, θ- and ι-CAs)^4–19^, therefore alternative, isoform selective CAI classes were searched. A multitude of new chemotypes as well as novel CA inhibition mechanisms were reported in the last decade^1–3^^,^^11–14^^,^^23–30^.

**Figure 1.**
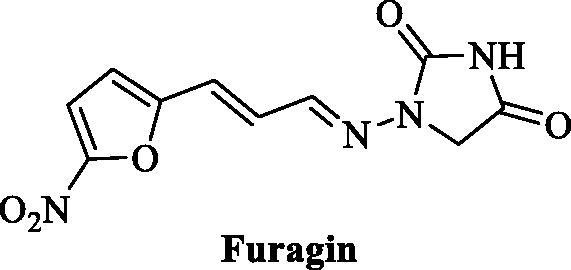
Structure of **Furagin**.

That has highly enriched our understanding of these enzymes and also allowed obtaining of isoform-selective CAIs targeting physiologically relevant isoforms^11–14^^,^^23–27^. Among the new chemotypes, which also exhibited the highest levels of isoform selectivity, were the coumarins^27^, the sulfocoumarins^23–26^ and their congeners, homosulfocoumarins (3H-1,2-benzoxathiepine 2,2-dioxides)^31^, and saccharin derivatives^32–34^. Considering the fact that this last chemotype was somewhat chemically similar to hydantoin (imidazolidine-2,4-dione) that may serve as zinc binding group (ZBG) we investigated clinically used antibiotic **Furagin** (Figure 1), also known under names Furazidine, Furamags or Furazidin^35^, that contains hydantoin moiety, as well as newly prepared its derivatives.

## 2. Materials and methods

### 2.1. Chemical syntheses – general

Reagents, starting materials and solvents were obtained from commercial sources and used as received. Thin-layer chromatography was performed on silica gel, spots were visualised with UV light (254 and 365 nm). Melting points were determined on an OptiMelt automated melting point system. IR spectra were recorded on Shimadzu FTIR IR Prestige-21 spectrometer. NMR spectra were recorded on Bruker Avance Neo (400 MHz) spectrometer with chemical shifts values (δ) in ppm relative to TMS using the residual DMSO-d_6_ signal (^1^H 2.50; ^13^C 39.52) or CDCl_3_ signal (^1^H 7.26; ^13^C 77.16) as an internal standard, or D_2_O signal and dioxane (^1^H 4.79; ^13^C 67.19). High-resolution mass spectra (HRMS) were recorded on a mass spectrometer with a Q-TOF micro mass analyser using the ESI technique. Examples of spectral data are furnished in the Supporting Information to the aricle.

### 2.2. General procedure for compound 2–17 synthesis

To a solution of 1-aminoimidazolidine-2,4-dione hydrochloride (**1**) (1.0 eq.) in EtOH (15 ml per 1 mmol of compound **1**) appropriate aldehyde (1.05 eq.) was added. The resulting mixture was stirred at room temperature overnight.

The solvent was removed under vacuum and the crude product was re-crystallized form EtOH to afford product.

#### 2.2.1. 1-(Benzylideneamino)-imidazolidine-2,4-dione (2)^36^


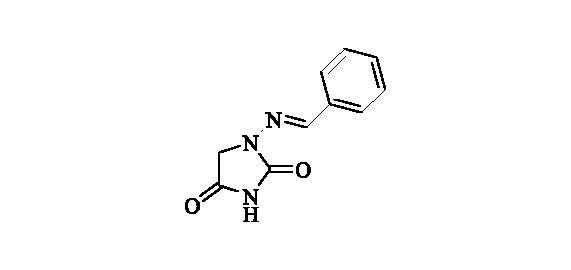
Compound **2** was prepared according to the general procedure from compound **1** (0.5 g; 3.30 mmol) and benzaldeyde (0.35 ml; 3.46 mmol) as white solid (0.60 g; 90%). Mp 252 – 253 °C. IR (film, cm^−1^) *ν*_max_= 1778 (C=O), 1717 (C=O); ^1^H NMR (400 MHz, DMSO-d_6_) *δ* = 4.36 (*s*, 2H), 7.38–7.48 (*m*, 3H), 7.68–7.72 (*m*, 2H), 7.80 (*s*, 1H), 11.25 (*s*, 1H) ppm ^13^C NMR (100 MHz, DMSO-d_6_) *δ* = 48.9, 126.8, 128.8, 129.8, 134.3, 143.0, 153.4, 169.0 ppm HRMS (ESI) [M + H]^+^: *m*/*z* calcd for (C_10_H_10_N_3_O_2_) 204.0773. Found 204.0783.

#### 2.2.2. 1-((4-Methoxybenzylidene)amino)imidazolidine-2,4-dione (3)


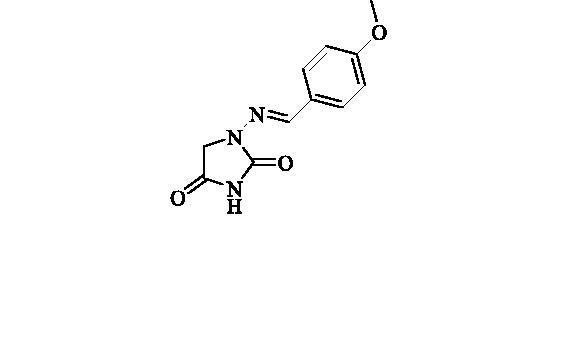
 Compound **3** was prepared according to the general procedure from compound **1** (0.5 g; 3.30 mmol) and 4-methoxybenzaldehyde (0.42 ml; 3.46 mmol) as white solid (0.62 g; 80%). Mp 242 – 244 °C. IR (film, cm^−1^) *ν*_max_= 1768 (C=O), 1718 (C=O); ^1^H NMR (400 MHz, DMSO-d_6_) *δ* = 3.80 (*s*, 3H), 4.33 (*s*, 2H), 6.99–7.04(*m*, 2H), 7.62–7.66 (*m*, 2H), 7.75 (*s*, 1H), 11.18 (*s*, 1H) ppm ^13^C NMR (100 MHz, DMSO-d_6_) *δ* = 48.9, 55.3, 114.3, 126.9, 128.4, 142.9, 153.4, 160.6, 169.1 ppm HRMS (ESI) [M + H]^+^: *m*/*z* calcd for (C_11_H_12_N_3_O_3_) 234.0879. Found 234.0885.

#### 2.2.3. 1-((4-Nitrobenzylidene)amino)imidazolidine-2,4-dione (4)^37^


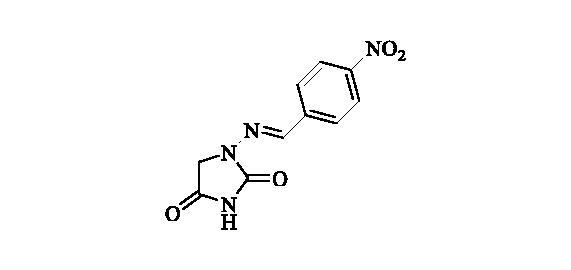
Compound **4** was prepared according to the general procedure from compound **1** (0.5 g; 3.30 mmol) and 4-nitrobenzaldehyde (0.52 g; 3.46 mmol) as yellowish solid (0.68 g; 82%). Mp 280 °C dec. IR (film, cm^−1^) *ν*_max_= 1780 (C=O), 1714 (C=O); ^1^H NMR (400 MHz, DMSO-d_6_) *δ* = 4.38 (*s*, 2H), 7.90–7.96 (*m*, 3H), 8.28–8.33 (*m*, 2H), 11.39 (*s*, 1H) ppm ^13^C NMR (100 MHz, DMSO-d_6_) *δ* = 49.1, 124.2, 127.7, 140.6, 140.7, 147.6, 153.4, 168.9 ppm HRMS (ESI) [M + H]^+^: *m*/*z* calcd for (C_10_H_9_N_4_O_4_) 249.0624. Found 249.0616.

#### 2.2.4. Methyl 4-(((2,4-dioxoimidazolidin-1-yl)imino)methyl)benzoate (5)


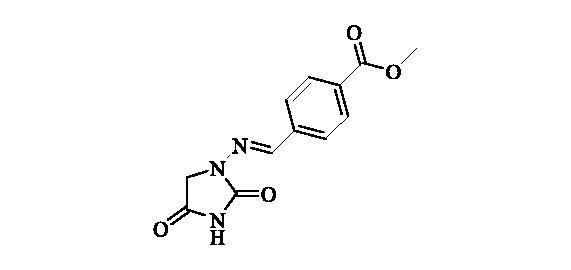
Compound **5** was prepared according to the general procedure from compound **1** (0.5 g; 3.30 mmol) and methyl 4-formylbenzoate (0.57 g; 3.46 mmol) as white solid (0.82 g; 95%). Mp 280 °C dec. IR (film, cm^−1^) *ν*_max_= 1763 (C=O), 1717 (C=O); ^1^H NMR (400 MHz, DMSO-d_6_) *δ* = 3.86 (*s*, 3H), 4.37 (*s*, 2H), 7.80–7.87 (*m*, 3H), 8.00–8.05 (*m*, 2H), 11.33 (*s*, 1H) ppm ^13^C NMR (100 MHz, DMSO-d_6_) *δ* = 49.0, 52.2, 127.0, 129.7, 130.2, 138.8, 141.6, 153.4, 165.9, 168.9 ppm HRMS (ESI) [M + H]^+^: *m*/*z* calcd for (C_12_H_12_N_3_O_4_) 262.0828. Found 262.0834.

#### 2.2.5. 1,1’-((Pentane-1,5-diylidene)bis(azaneylylidene))bis(imidazolidine-2,4-dione) (6)


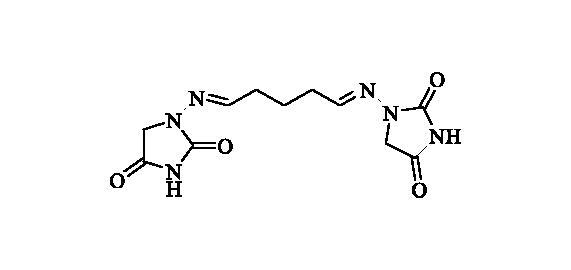
Compound **6** was prepared according to the general procedure from compound **1** (0.5 g; 3.30 mmol) and glutaraldehyde 50 wt % solution in H_2_O (0.31 ml; 3.46 mmol) as white solid (0.49 g; 50%). Mp 237 °C dec. IR (film, cm^−1^) *ν*_max_= 1768 (C=O), 1734 (C=O); ^1^H NMR (400 MHz, DMSO-d_6_) *δ* = 1.72 (p, 2H, *J* = 7.4 Hz), 2.28–2.36 (*m*, 4H), 4.17 (*s*, 4H), 7.06 (*t*, 2H, *J* = 5.2 Hz), 11.07 (*s*, 2H) ppm ^13^C NMR (100 MHz, DMSO-d_6_) *δ* = 23.1, 31.3, 48.5, 146.7, 153.4, 169.1 ppm HRMS (ESI) [M + Na]^+^: *m*/*z* calcd for (C_11_H_14_N_6_O_4_Na) 317.0974. Found 317.0978.

#### 2.2.6. 1-((Furan-3-ylmethylene)amino)imidazolidine-2,4-dione (7)


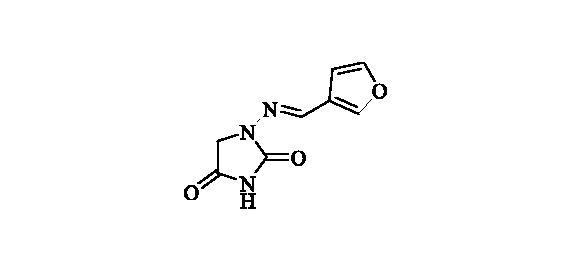
Compound **7** was prepared according to the general procedure from compound **1** (0.5 g; 3.30 mmol) and 3-furaldehyde (0.33 g; 3.46 mmol) as yellowish solid (0.57 g; 89%). Mp 235 °C dec. IR (film, cm^−1^) *ν*_max_= 1780 (C=O), 1714 (C=O);

^1^H NMR (400 MHz, DMSO-d_6_) *δ* = 4.30 (s, 2H), 6.74–6.76 (*m*, 1H), 7.73–7.77 (*m*, 2H), 8.05–8.07 (*m*, 1H), 11.18 (*s*, 1H) ppm ^13^C NMR (100 MHz, DMSO-d_6_) *δ* = 48.8, 107.0, 122.5, 136.1, 144.8, 144.9, 153.3, 169.1 ppm HRMS (ESI) [M + H]^+^: *m*/*z* calcd for (C_8_H_8_N_3_O_3_) 194.0566. Found 194.0570.

#### 2.2.7. 1-((4-(Benzyloxy)benzylidene)amino)imidazolidine-2,4-dione (8)


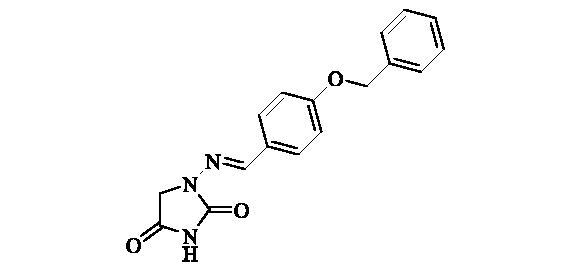
Compound **8** was prepared according to the general procedure from compound **1** (0.5 g; 3.30 mmol) and 4-benzyloxybenzaldehyde (0.73 g; 3.46 mmol) as white solid (0.92 g; 90%). Mp 258–260 °C. IR (film, cm^−1^) *ν*_max_= 1790 (C=O), 1730 (C=O); ^1^H NMR (400 MHz, DMSO-d_6_) *δ* = 4.33 (*s*, 2H), 5.15 (*s*, 2H), 7.07–7.12 (*m*, 2H), 7.31–7.36 (*m*, 1H), 7.37–7.43 (*m*, 2H), 7.44–7.49 (*m*, 2H), 7.62–7.67 (*m*, 2H), 7.75 (*s*, 1H), 11.19 (*s*, 1H) ppm ^13^C NMR (100 MHz, DMSO-d_6_) *δ* = 48.9, 69.4, 115.1, 127.1, 127.8, 127.9, 128.4, 128.5, 136.8, 142.8, 153.4, 159.7, 169.1 ppm HRMS (ESI) [M + H]^+^: *m*/*z* calcd for (C_17_H_16_N_3_O_3_) 310.1192. Found 310.1194.

#### 2.2.8. Ethyl (2E)-4-((2,4-dioxoimidazolidin-1-yl)imino)but-2-enoate (9)


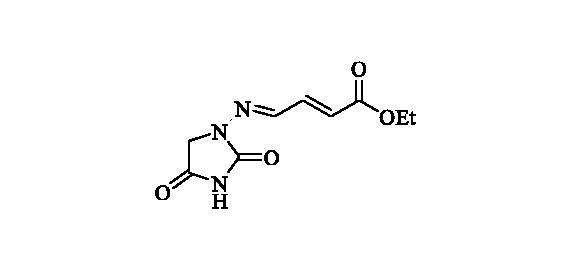
Compound **9** was prepared according to the general procedure from compound **1** (0.5 g; 3.30 mmol) and ethyl trans-4-oxo-2-butenoate (0.42 ml; 3.46 mmol) as white solid (0.60 g; 81%). Mp 210–211 °C. IR (film, cm^−1^) *ν*_max_= 1772 (C=O), 1721 (C=O); ^1^H NMR (400 MHz, DMSO-d_6_) *δ* = 1.24 (*t*, 3H, *J* = 7.1 Hz), 4.17 (*q*, 2H, *J* = 7.1 Hz), 4.27 (*s*, 2H), 7.37 (*d*, 1H, *J* = 15.6 Hz), 7.16–7.24 (*m*, 1H), 7.60 (*d*, 1H, *J* = 9.3 Hz), 11.39 (*s*, 1H) ppm ^13^C NMR (100 MHz, DMSO-d_6_) *δ* = 14.1, 49.0, 60.4, 126.5, 140.3, 141.7, 153.2, 165.4, 168.7 ppm HRMS (ESI) [M + H]^+^: *m*/*z* calcd for (C_9_H_12_N_3_O_4_) 226.0828. Found 226.0834.

#### 2.2.9. 1-((3-Methylbut-2-en-1-ylidene)amino)imidazolidine-2,4-dione (10)


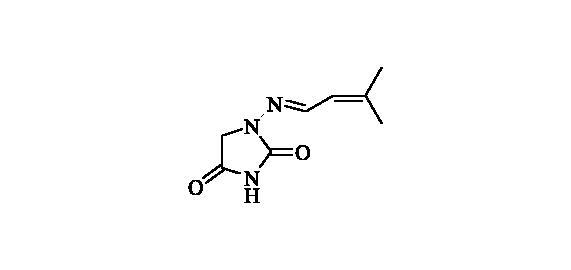
Compound **10** was prepared according to the general procedure from compound **1** (0.5 g; 3.30 mmol) and 3-methyl-2-butenal (0.33 ml; 3.46 mmol) as white solid (0.43 g; 72%). Mp 186–187 °C. IR (film, cm^−1^) *ν*_max_= 1768 (C=O), 1717 (C=O); ^1^H NMR (400 MHz, DMSO-d_6_) *δ* = 1.84–1.89 (*m*, 6H), 4.28 (*s*, 2H), 5.93–5.99 (*m*, 1H), 7.57 (d, 1H, *J* = 9.5 Hz), 11.11 (*s*, 1H) ppm ^13^C NMR (100 MHz, DMSO-d_6_) *δ* = 18.7, 26.2, 48.9, 121.9, 142.4, 144.3, 153.3, 169.2 ppm HRMS (ESI) [M + H]^+^: *m*/*z* calcd for (C_8_H_12_N_3_O_2_) 182.0930. Found 182.0938.

#### 2.2.10 1-(((2e)-3–(4-methoxyphenyl)allylidene)amino)imidazolidine-2,4-dione (11)


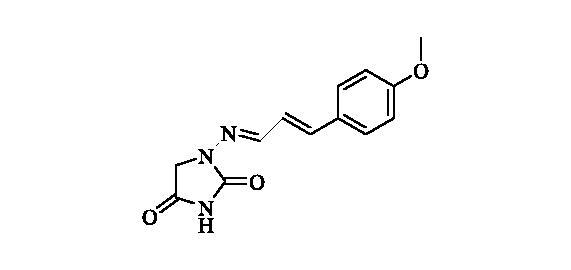
Compound **11** was prepared according to the general procedure from compound **1** (0.5 g; 3.30 mmol) and trans-4-methoxycinnamaldehyde (0.56 g; 3.46 mmol) as white solid (0.61 g; 71%). Mp 250 °C dec. IR (film, cm^−1^) *ν*_max_= 1770 (C=O), 1731 (C=O); ^1^H NMR (400 MHz, DMSO-d_6_) *δ* = 3.78 (*s*, 3H), 4.29 (*s*, 2H), 6.85–7.00 (*m*, 4H), 7.51–7.59 (*m*, 3H), 11.18 (*s*, 1H) ppm ^13^C NMR (100 MHz, DMSO-d_6_) *δ* = 48.8, 55.2, 114.3, 123.1, 128.5, 128.6, 138.5, 145.5, 153.3, 159.9, 169.1 ppm HRMS (ESI) [M + H]^+^: *m*/*z* calcd for (C_13_H_14_N_3_O_3_) 260.1035. Found 260.1047.

#### 2.2.11. 1-((2,4-Dihydroxybenzylidene)amino)imidazolidine-2,4-dione (12)


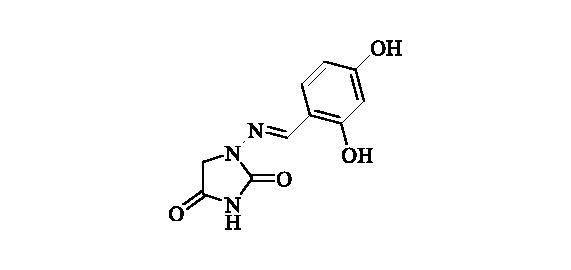
Compound **12** was prepared according to the general procedure from compound **1** (0.5 g; 3.30 mmol) and 2,4-dihydroxybenzaldehyde (0.48 g; 3.46 mmol) as white solid (0.72 g; 93%). Mp >300 °C. IR (film, cm^−1^) *ν*_max_= 3260 (OH), 3188 (OH), 1780 (C=O), 1717 (C=O); ^1^H NMR (400 MHz, DMSO-d_6_) *δ* = 4.34 (*s*, 2H), 6.31 (d, 1H, *J* = 2.3 Hz), 6.35 (dd, 1H, *J* = 8.5, 2.3 Hz), 7.33 (d, 1H, *J* = 8.5 Hz), 7.90 (*s*, 1H), 9.90 (br s, 1H), 10.73 (*s*, 1H), 11.23 (br s, 1H) ppm ^13^C NMR (100 MHz, DMSO-d_6_) *δ* = 48.5, 102.6, 107.8, 110.7, 130.5, 144.0, 153.3, 158.6, 160.5, 169.1 ppm HRMS (ESI) [M + H]^+^: *m*/*z* calcd for (C_10_H_10_N_3_O_4_) 236.0671. Found 236.0677.

#### 2.2.12. 4-(((2,4-Dioxoimidazolidin-1-yl)imino)methyl)phenyl)boronic acid (13)


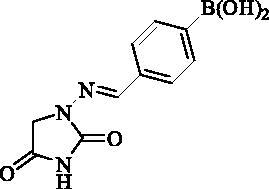
Compound **13** was prepared according to the general procedure from compound **1** (0.5 g; 3.30 mmol) and 4-formylphenylboronic acid (0.52 g; 3.46 mmol) as white solid (0.72 g; 88%). Mp >300 °C. IR (film, cm^−1^) *ν*_max_= 3349 (OH), 3173 (OH), 1780 (C=O), 1716 (C=O); ^1^H NMR (400 MHz, DMSO-d_6_) *δ* = 4.37 (s, 2H), 7.64–7.68 (*m*, 2H), 7.79 (*s*, 1H), 7.83–7.87 (*m*, 2H), 8.12 (*s*, 2H), 11.26 (*s*, 1H) ppm ^13^C NMR (100 MHz, DMSO-d_6_) *δ* = 48.9, 125.8, 134.5, 135.7, 136.0 (br) 143.0, 153.4, 169.1 ppm HRMS (ESI) [M + H]^+^: *m*/*z* calcd for (C_10_H_11_BN_3_O_4_) 248.0843. Found 248.0847.

#### 2.2.13. 1-((Pyridin-2-ylmethylene)amino)imidazolidine-2,4-dione (14)


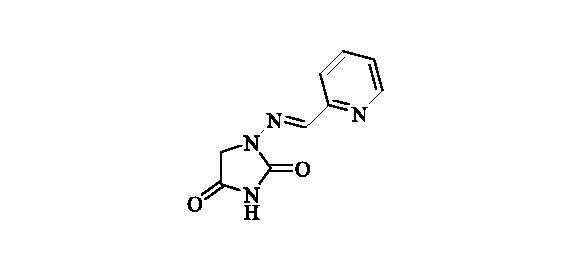
Compound **14** was prepared according to the general procedure from compound **1** (0.5 g; 3.30 mmol) and pyridine-2-carbaldehyde (0.33 ml; 3.46 mmol) as white solid (0.64 g; 95%). Mp 280 °C dec. IR (film, cm^−1^) *ν*_max_= 1770 (C=O), 1730 (C=O); ^1^H NMR (400 MHz, DMSO-d_6_) *δ* = 4.43 (*s*, 2H), 7.61–7.66 (*m*, 1H), 7.89 (*s*, 1H), 8.02–8.06 (*m*, 1H), 8.16 (dt, 1H, *J* = 7.7, 1.4 Hz), 8.68–8.72 (*m*, 1H), 11.50 (*s*, 1H) ppm ^13^C NMR (100 MHz, DMSO-d_6_) *δ* = 49.0, 121.6, 125.2, 139.3, 140.6, 146.8, 150.5, 153.3, 168.7 ppm HRMS (ESI) [M + H]^+^: *m*/*z* calcd for (C_9_H_9_N_4_O_2_) 205.0726. Found 205.0732.

#### 2.2.14. 1-((Pyridin-3-ylmethylene)amino)imidazolidine-2,4-dione (15)


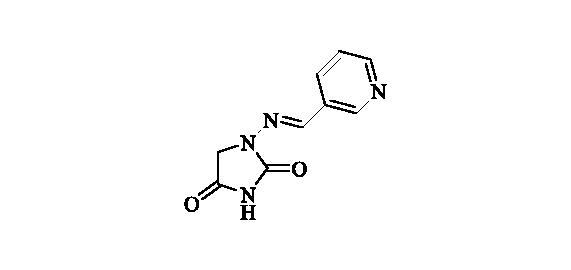
Compound **15** was prepared according to the general procedure from compound **1** (0.5 g; 3.30 mmol) and pyridine-3-carbaldehyde (0.33 ml; 3.46 mmol) as white solid (0.60 g; 90%). Mp 280 °C dec. IR (film, cm^−1^) *ν*_max_= 1764 (C=O), 1722 (C=O); ^1^H NMR (400 MHz, D_2_O + NaOH + dioxane) *δ* = 7.46–7.51 (*m*, 1H), 7.56 (*s*, 1H), 8.18 (td, 1H, *J* = 8.0, 1.8 Hz), 8.48 (dd, 1H, *J* = 4.9, 1.6 Hz), 8.74 (d, 1H, *J* = 1.8 Hz) ppm ^13^C NMR (100 MHz, D_2_O + NaOH + dioxane) *δ* = 49.3 (br), 125.1, 131.6, 135.1, 138.9, 148.1, 149.7, 170.0, 186.3 ppm HRMS (ESI) [M + H]^+^: *m*/*z* calcd for (C_9_H_9_N_4_O_2_) 205.0726. Found 205.0731.

#### 2.2.15. 1-((Pyridin-4-ylmethylene)amino)imidazolidine-2,4-dione (16)


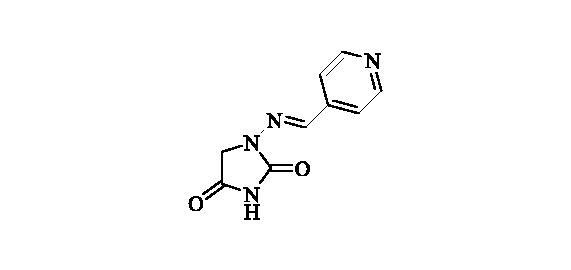
Compound **16** was prepared according to the general procedure from compound **1** (0.5 g; 3.30 mmol) and pyridine-4-carbaldehyde (0.33 ml; 3.46 mmol) as white solid (0.61 g; 91%). Mp 280 °C dec. IR (film, cm^−1^) *ν*_max_= 1750 (C=O), 1723 (C=O); ^1^H NMR (400 MHz, D_2_O + NaOH + dioxane) *δ* = 7.46 (*s*, 1H), 7.62–7.66 (*m*, 2H), 8.47–8.51 (*m*, 2H) ppm ^13^C NMR (100 MHz, D_2_O + NaOH + dioxane) *δ* = 49.3 (br), 121.9, 139.2, 143.5, 149.7, 170.0, 186.4 ppm HRMS (ESI) [M + H]^+^: *m*/*z* calcd for (C_9_H_9_N_4_O_2_) 205.0726. Found 205.0730.

#### 2.2.16. 1-(((1 h-Imidazol-5-yl)methylene)amino)imidazolidine-2,4-dione (17)


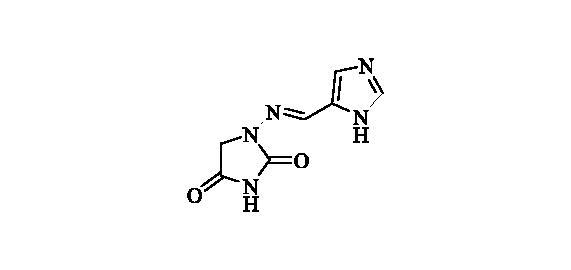
Compound **17** was prepared according to the general procedure from compound **1** (0.5 g; 3.30 mmol) and 1H-imidazole-5-carbaldehyde (0.33 g; 3.46 mmol) as white solid (0.62 g; 97%). Mp 270 °C dec. IR (film, cm^−1^) *ν*_max_= 1764 (C=O), 1715 (C=O); ^1^H NMR (400 MHz, D_2_O + NaOH + dioxane) *δ* = 7.45–7.48 (*m*, 1H), 7.67 (*s*, 1H), 7.72–7.76 (*m*, 1H) ppm ^13^C NMR (100 MHz, D_2_O + NaOH + dioxane) *δ* = 49.5 (br), 125.1, 134.2, 136.5, 140.8, 170.3, 186.7 ppm HRMS (ESI) [M + H]^+^: *m*/*z* calcd for (C_7_H_8_N_5_O_2_) 194.0678. Found 194.0687

### 2.3. Ca inhibitory assay

An Applied Photophysics stopped-flow instrument has been used for assaying the CA catalysed CO2 hydration activity, as reported earlier^38^^,^^39^. The inhibition constants were obtained by non-linear least-squares methods using PRISM 3 and the Cheng-Prusoff equation as reported earlier^40^ and represent the mean from at least three different determinations. The four tested CA isoforms were recombinant ones obtained in-house as reported earlier^41–43^.

### 2.4. Computational studies

The crystal structure of CA II (pdb 5LJT)^43^, CA IX (pdb 5FL4)^44^ and CA XII (pdb JLD0)^45^ were prepared using the Protein Preparation Wizard tool implemented in Maestro - Schrödinger suite, assigning bond orders, adding hydrogens, deleting water molecules, and optimising H-bonding networks^46^. Energy minimisation protocol with a root mean square deviation (RMSD) value of 0.30 was applied using an Optimised Potentials for Liquid Simulation (OPLS3e) force field. 3D ligand structures were prepared by Maestro^46^^a^ and evaluated for their ionisation states at pH 7.4 ± 0.5 with Epik^46^^b^. Additionally, the imidic nitrogen of the hydantoin nucleus was negatively charged in simulations. OPLS3e force field in Macromodel^46^^e^ was used for energy minimisation for a maximum number of 2500 conjugate gradient iteration and setting a convergence criterion of 0.05 kcal mol^−1 ^Å^−1^. The docking grid was centred on the centre of mass of the co-crystallized ligands and Glide used with default settings. Ligands were docked with the standard precision mode (SP) of Glide^46^^e^ and the best 5 poses of each molecule retained as output. The best pose for each compound, evaluated in terms of coordination, hydrogen bond interactions and hydrophobic contacts, was refined with Prime^46^^d^ with a VSGB solvation model considering the target flexible within 3 Å around the ligand^47–49^.

The best poses of Furagin and **12** to CA II, CA IX and CA XII were submitted to a MD simulation using Desmond^50^ and the OPL3e force field. Specifically, the system was solvated in an orthorhombic box using TIP4PEW water molecules, extended 15 Å away from any protein atom. It was neutralised adding chlorine and sodium ions. The simulation protocol included a starting relaxation step followed by a final production phase of 100 ns. In particular, the relaxation step comprised the following: (a) a stage of 100 ps at 10 K retaining the harmonic restraints on the solute heavy atoms (force constant of 50.0 kcal mol^−1 ^Å^−2^) using the NPT ensemble with Brownian dynamics; (b) a stage of 12 ps at 10 K with harmonic restraints on the solute heavy atoms (force constant of 50.0 kcal mol^−1 ^Å^−2^), using the NVT ensemble and Berendsen thermostat; (c) a stage of 12 ps at 10 K and 1 atm, retaining the harmonic restraints and using the NPT ensemble and Berendsen thermostat and barostat; (f) a stage of 12 ps at 300 K and 1 atm, retaining the harmonic restraints and using the NPT ensemble and Berendsen thermostat and barostat; (g) a final 24 ps stage at 300 K and 1 atm without harmonic restraints, using the NPT Berendsen thermostat and barostat. The final production phase of MD was run using a canonical NPT Berendsen ensemble at temperature 300 K. During the MD simulation, a time step of 2 fs was used while constraining the bond lengths of hydrogen atoms with the M-SHAKE algorithm. The atomic coordinates of the system were saved every 100 ps along the MD trajectory. Protein and ligand RMSD values, ligand torsions evolution and occupancy of intermolecular hydrogen bonds and hydrophobic contacts were computed along the production phase of the MD simulation with the Simulation Interaction Diagram tools implemented in Maestro.

## 3. Results and discussion

### 3.1. Chemistry

**Scheme 1.**
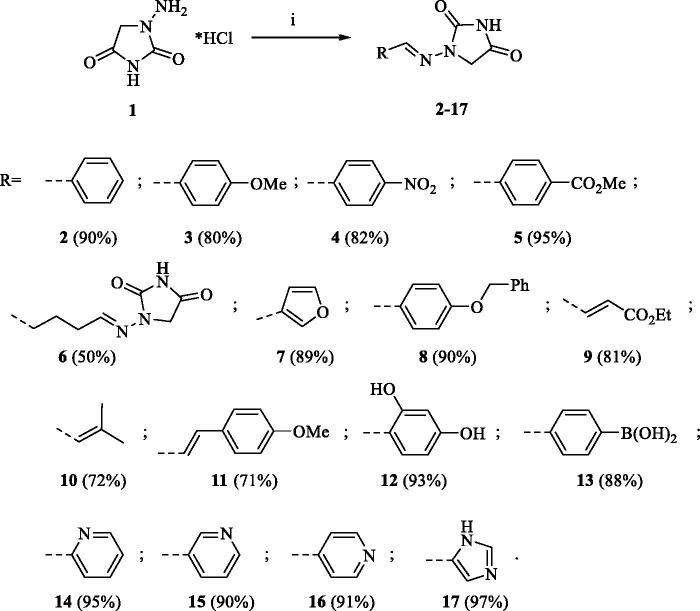
Reagents and conditions: i. RCHO, EtOH, RT, 16 h

A series of Furagin derivatives **2**–**17** were prepared in reaction between 1-aminohydantoin hydrochloride (**1**) and various aldehydes (Scheme 1). Compounds **2–17** were isolated in good to excellent yields, all new structure were proven by ^1^H and ^13^C NMR and IS spectroscopy as well as high-resolution mass spectra. The purity of all compounds was greater than 95% according UPLC analysis.

### 3.2. Biological evaluation

The CA inhibitory profiles of Furagin and synthesised aminohydantoin derivatives were evaluated by applying a stopped flow carbon dioxide hydrase assay^51^, in comparison to acetazolamide (AAZ) as a standard CAI against four physiologically significant isoforms CA I, II, IX, and XII. The following structure–activity relationship (SAR) can be concluded from the inhibition data presented in Table 1.

**Table 1.** Inhibition data of human CA isoforms CA I, II, IX, and XII with aminohydantoines (**2**–**17**, Furagin) using **AAZ** as a standard inhibitor.
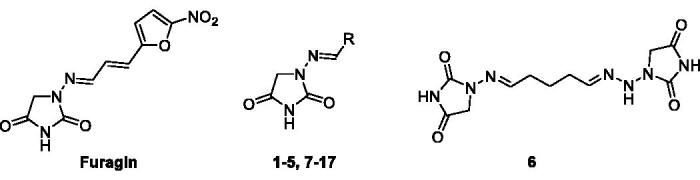

Comp.	R	*K*_I_ (nM)*			
		CA I	CA II	CA IX	CA XII
**2**	C_6_H_5_	39 600	900	3500	5600
**3**	4-OCH_3_-C_6_H_4_	57 600	6400	1200	4700
**4**	4-NO_2_-C_6_H_4_	>100 000	11 100	7400	2800
**5**	4-(CO_2_CH_3_)-C_6_H_4_	>100 000	8300	4900	930
**6**	**-**	19 100	4000	1100	160
**7**	3-furanyl	16 800	710	850	1700
**8**	4-(OCH_2_C_6_H_5_)-C_6_H_4_	>100 000	540	350	910
**9**	CHCH(CO_2_C_2_H_5_)	45 900	23 600	810	440
**10**	CHC(CH_3_)_2_	28 800	16 500	2900	880
**11**	CHCH(4-OCH_3_-C_6_H_4_)	>100 000	3100	400	360
**12**	2,4-(OH)_2_-C_6_H_3_	>100 000	59 900	5800	150
**13**	4-(B(OH)_2_)-C_6_H_4_	90 700	14 200	7300	230
**14**	2-pyridyl	51 800	4200	4500	1300
**15**	3-pyridyl	45 600	620	2300	3200
**16**	4-pyridyl	26 600	3300	1600	810
**17**	5-imidazolyl	9600	12 400	560	350
**Furagin**	**-**	>100 000	9600	260	57
**AAZ**	**-**	250	12	25	6

*Mean from 3 different assays, by a stopped flow technique (errors were in the range of ± 5–10% of the reported values).

- All the tested aminohydantoins exhibited weak inhibitory effect on the slow cytosolic isoform, hCA I, where the binding affinity constant (*K*_I_) values fluctuating in the thousands nM range (*K*_I_ 16 800->100 000 nM).
- The physiologically relevant isoform, hCA II, was better inhibited by most of the tested compounds (*K*_I_s: 620-59 000 nM). It is observed that, the aminohydantoin compounds (**2**, **7**, **8** and **15**) were more potent hCA II inhibitors with *K*_I_s in range from 540-900 nM. These compounds have unsubstituted Ph or hetaryl moieties. Rest of the compounds showed weaker inhibitory effect of CA II with *K*_I_s in range from 3100–59 900 nM. It is interesting to note, that compound **12** having dihydroxyphenyl substituent stood out by nearly three times weaker inhibition compare to the second weakest inhibitor **9**.
- The tumour associated isoform hCA IX was inhibited in nano-molar range by compounds **7**-**9**, **11**, **17** and Furagin (*K*_I_s: 260–850 nM), where the strongest inhibition was observed for Furagin. Rest of the aminohydantoin derivatives showed one order weaker inhibition with *K*_I_s in range from 1100-7 300 nM. Certain pattern can be observed, where better CA IX inhibition can be observed for compounds with vinyl substituents (**9**, **11**, **17** and Furagin) or small hetaryl substituents (**7** and **17**), with exception in case of compound **8**, containing ester moiety on phenyl ring.
- The other tumour associated isoform hCA XII was best inhibited among all isoforms studied. The best compound of this series was Furagin with *K*_I_ = 57 nM. It was followed by vinyl substituted aminohydantoin derivatives **6**, **9** and **10** with *K*_I_s 160, 360 and 880 nM, respectively. One order weaker CA XII inhibition compare to Furagin was also observed for aryl (**5**, **8** and **12**) and hetaryl (**16** and **17**) derivatives ranging *K*_I_s from 150 to 930 nM.

In general good selectivity against cancer associated CA isoforms (CA IX and CA XII) compare to off-target ones (CA I and CA II) was observed for three compounds Furagin, **9** and **12**.

### 3.3. Computational studies

**Figure 2.**
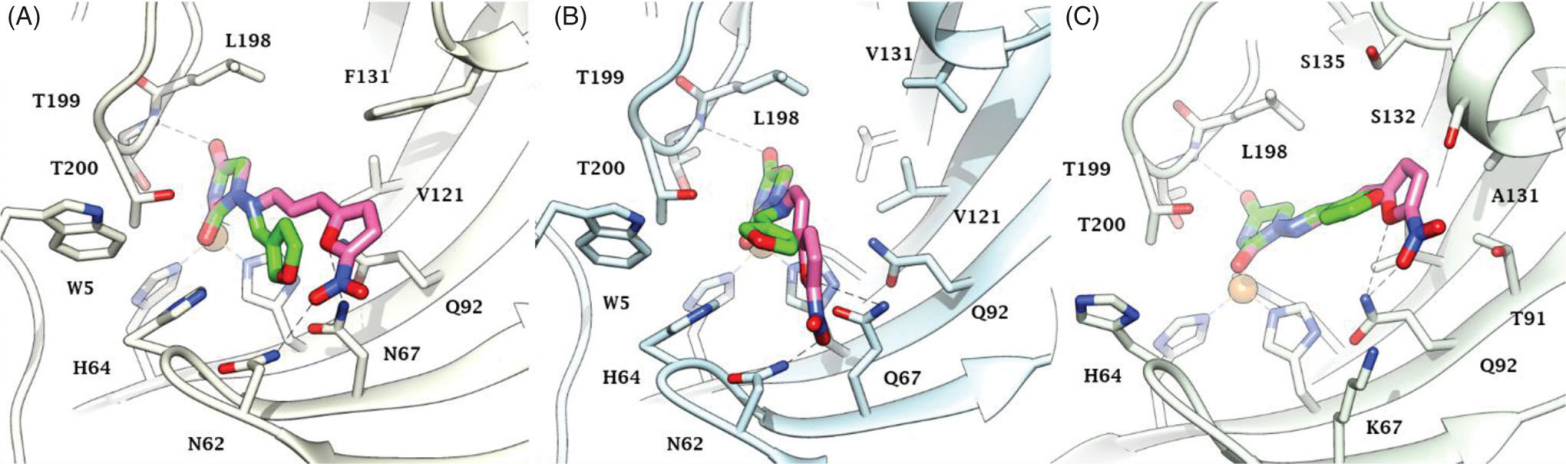
Predicted docking orientations of **7** (green) and **Furagin** (pink) to (A) CA II, (B) CA IX and (C) CA XII.

Docking studies were used to investigate the binding mode of Furagin and aminohydantoines **2-17** within the active site of CA II (pdb 5LJT)^44^, IX (pdb 5FL4)^43^ and XII (pdb JLD0)^45^. Similarly to benzenesulfonamides (pKa 10.1) which binds to the CA Zn ion in the deprotonated form, the imidic nitrogen of the hydantoin nucleus as well was considered negatively charged (pKa 9.16)^52^ in the docking experiments and resulted to coordinate the zinc ion in all the obtained poses with CAs II, IX and XII. Furthermore, the oxygen atom of the CO in position 4 of the hydantoin core acts as a bifurcated acceptor establishing two H-bonds with T199, that is, O^…^(H-N, HG1-O), whereas overall the heterocycle forms VdW contacts with residues H94, H96, H119, L198, T200 and W209 (Figure 2).

**Figure 3.**
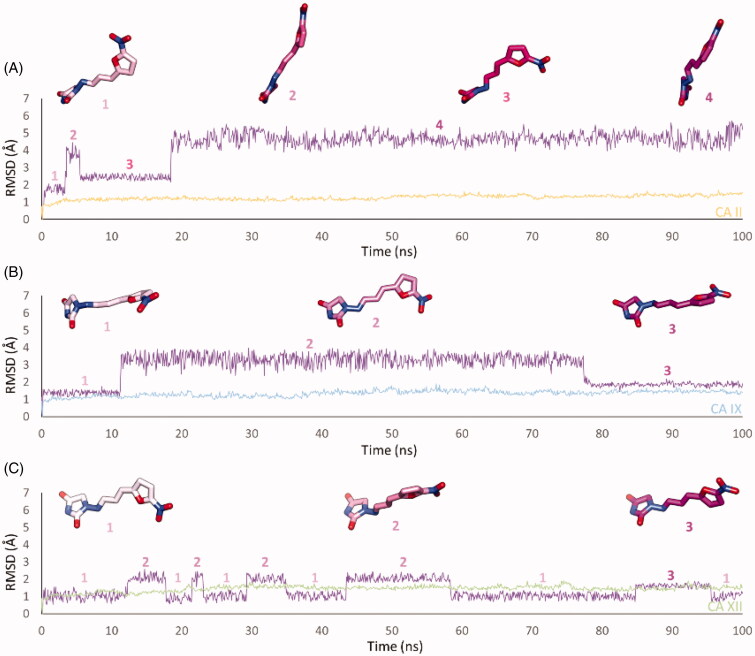
RMSD analysis of Furagin heavy atoms and (A) CA II, (B) CAIX and (C) CA XII backbone over the 100 ns MD simulation. The ligand colour darkens over the dynamic simulation.

**Figure 4.**
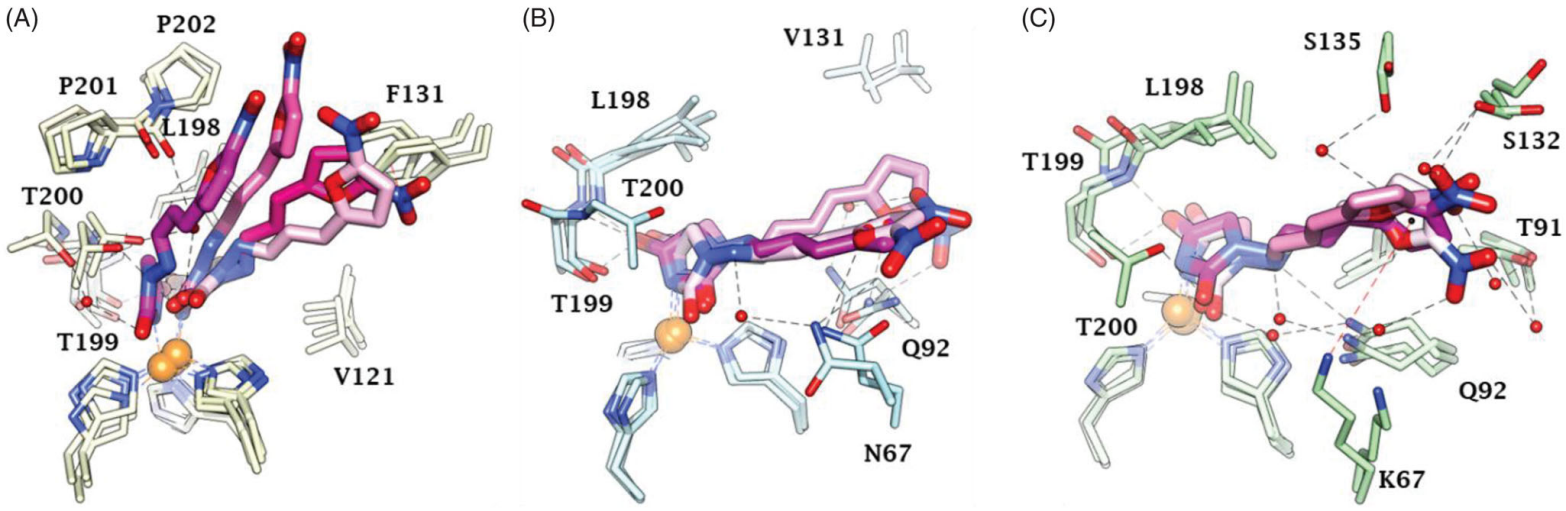
Dynamics evolution of the binding mode of Furagin to (A) CA II, (B) CA IX and (C) CA XII over the course of 100 ns. Water molecules are represented as red spheres. The ligand colour darkens over the dynamic simulation.

**Figure 5.**
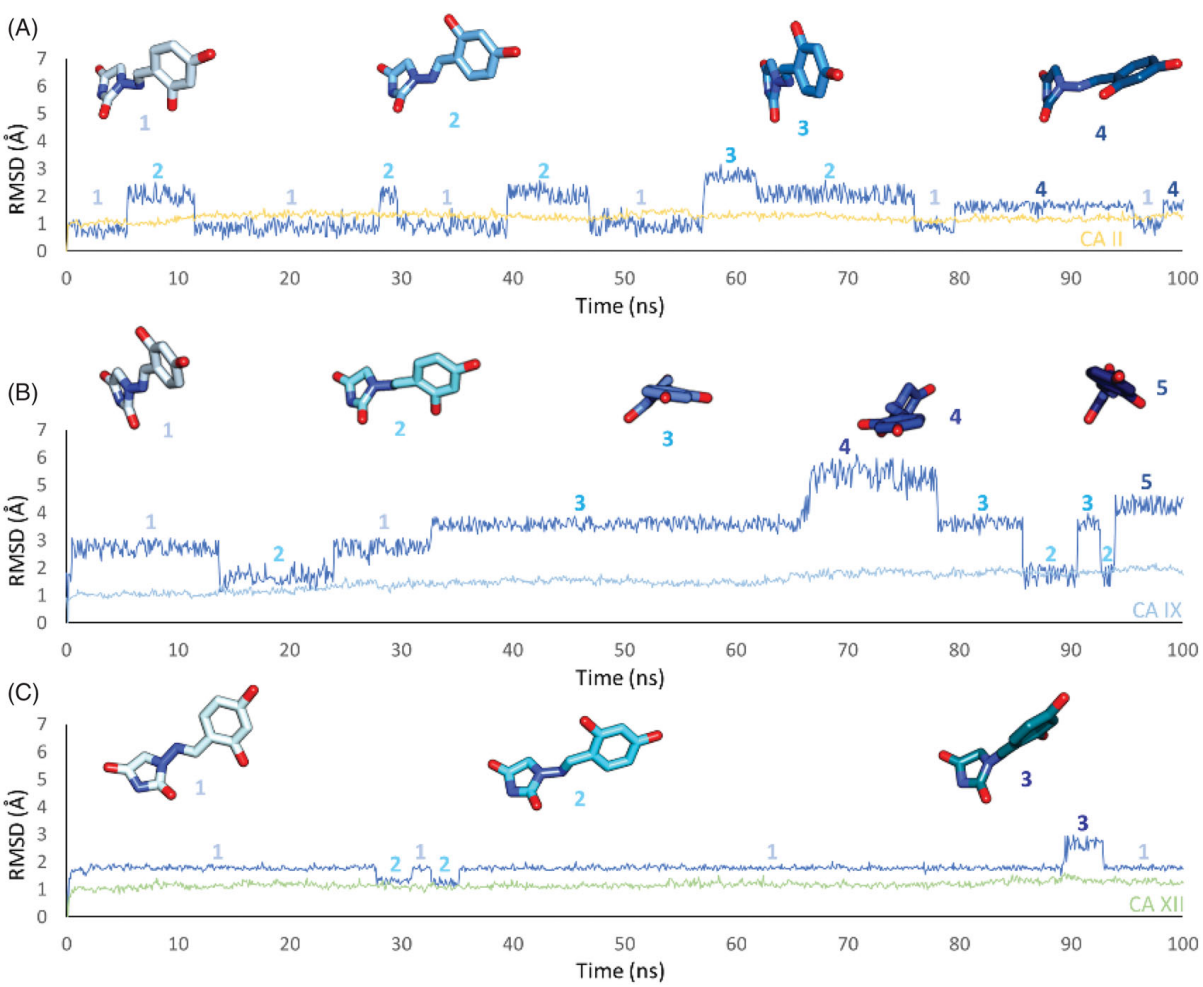
RMSD analysis of **12** heavy atoms and (A) CA II, (B) CAIX and (C) CA XII backbone over the 100 ns MD simulation. The ligand colour darkens over the dynamic simulation.

**Figure 6.**
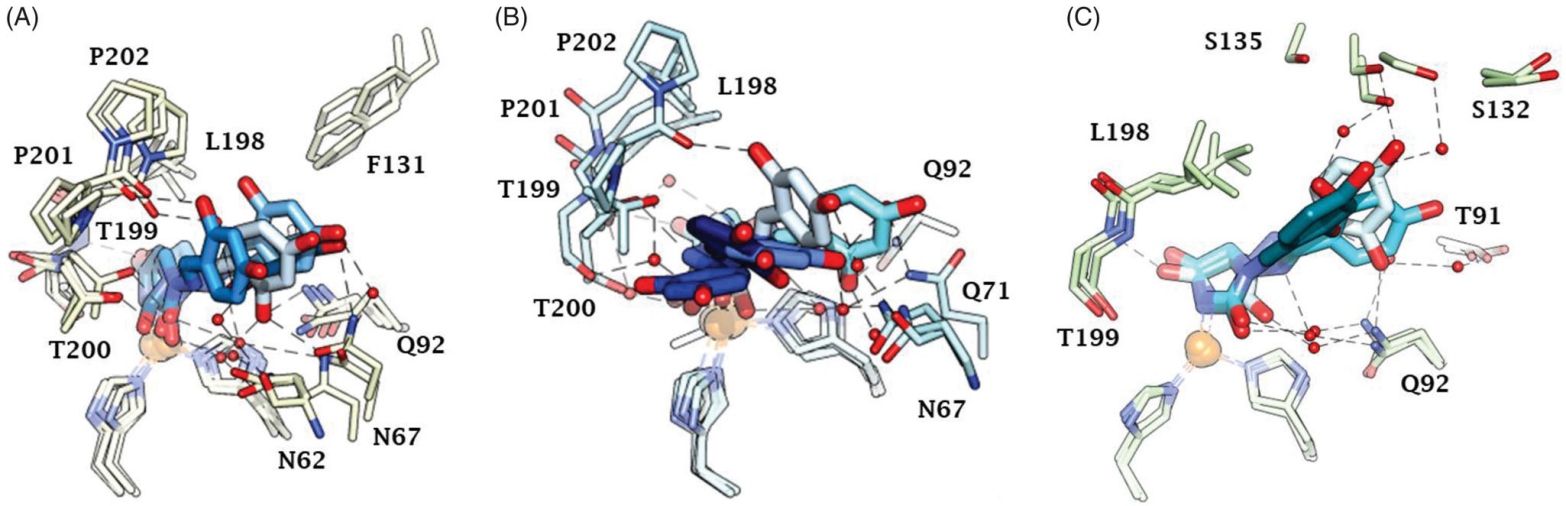
Dynamics evolution of the binding mode of **12** to (A) CA II, (B) CA IX and (C) CA XII over the course of 100 ns. Water molecules are represented as red spheres. The ligand colour darkens over the dynamic simulation.

In CA II and CA IX, the N_1_ pendants of all ligands are oriented towards a hydrophilic cleft defined by H4, W5, N62, N67 and H64, except **8** and **9**, whose N_1_ tails are housed, in CA II, into a hydrophobic pocket formed by I91, V121 and F131 (Figure 2(A–B)). Amino acids T91, Q92, A131, S132 and S135 are instead targeted by the pendants on the aminohydantoin of the ligands in all docking solutions with CA XII (Figure 2(C)). The docking procedure was complemented with 100 ns long molecular dynamic (MD) simulations on the predicted binding conformations of Furagin and **12**, the most potent CA XII inhibitors also showing significant CA XII over CA II selectivity. The structure of the three investigated CA isoforms was stable during the computation with the backbone atom RMSDs exhibiting small fluctuations over the 100 ns (Figures 3 and 5). Additionally, the ZBG of the ligands remains stably anchored to the metal ion all over the MD, with the hydantoin core receiving H-bonds by the amidic NH and side chain OH of Thr199 (Figures 4 and 6).

After an initial equilibration, mainly occurring in CA II and IX, the molecular tail of Furagin undergoes minor conformational fluctuations during simulation approaching to stable binding conformations within the three CA isoforms (Figures 3 and 4). In CA IX and XII, the ligand accommodates the N1-pendant in the hydrophilic half of the active sites where it makes VdW contacts and both direct and water mediated H-bond interactions with the enzymes (Figure 4(B,C)). In the CA II, the ligand-bound conformation of Furagin orients the tail towards the hydrophobic area of the target and does not form persistent H-bond interactions over the 100 ns (Figure 4(A)). The hydrogen bond persistence within the three CA isoforms is in good agreement with the inhibitory profile of the ligand (CAXII > CA IX > CA II).

An ensemble of few conformations is representative of the binding of **12** within CA II and IX (Figures 5 and 6). Here, the ligand approaches the hydrophobic regions of the enzymes and, coming next to the end of the simulation, the N_1_ tails lose direct or water-bridged H-bonds with glutamine and asparagine residues, progressively moving towards T199 or T200, that is, the area of the enzyme that undergoes to the greatest residue displacement. In CA XII, the docked pose of **12** remains firmly anchored to the residues of the hydrophilic portion of the enzyme throughout the dynamic. A wide network of direct and water mediated H-bonds stabilise the binding of the ligand. This is consistent with the inhibition profile exhibited by **12** in CA XII as compared with the other two CA isoforms.

## 4. Conclusions

In summary, we have demonstrated that clinically used antibiotic – Furagin and its derivatives **2**-**17** are potential CA inhibitors. Furagin and all newly synthesised hydantoin derivative were examined for their inhibitory activities towards hCA I, II, IX and XII. The four studied hCA isoforms were inhibited by Furagin and its derivatives at various degrees. In particular, Furagin and prepared compounds **2**-**17** did not inhibit or poorly inhibited off-target hCA I with *K*_I_s ranging from >100 μM (compounds **4**, **5**, **8**, **11**, **12** and Furagin) to 9.6 μM. Ubiquitous hCA II was poorly inhibited by compounds **3**-**6**, **9**-**14**, **16**, **17** and Furagin (*K*_I_s from 59.9 to 3.1 μM). Rest of the compounds significantly inhibited hCA II (*K*_I_s from 900 nM to 540 nM). Remarkable inhibition of cancer associated hCA IX was observed for Furagin (*K*_I_=260 nM) and compounds **7**–**9**, **11** and **17** with *K*_I_s ranging from 350 to 850 nM. The rest of compounds exhibited slightly weaker inhibition of hCA IX with *K*_I_s ranging from 1100 to 7400 nM. Other cancer associated isoform – hCA XII also was significantly inhibited by Furagin (*K*_I_=57 nM) and compounds **5**, **6**, **8**-**13**, **16** and **17** (*K*_I_s from 160 to 910 nM). The rest of the compounds exhibited slightly weaker inhibition with *K*_I_s ranging from 1300 to 5600 nM. Docking and molecular dynamics simulations shed light on the ligands selectivity for the cancer-associated CAs over ubiquitous CA II. The significant inhibition activity and especially selectivity of Furagin against hCA IX and XII was attributed due to the strong H-bond interactions, whereas in case of hCA II no persistent H-bond interactions are formed due to Furagin’s tails orientation towards hydrophobic area of the enzyme.

The knowledge obtained gives the solid base for both – investigation of drug repurposing of clinically used antibiotic Furagin for anti-cancer therapy and further studies of new chemotype of inhibitors of CAs.

## Supplementary Material

Supplemental Material
