# Supplementary material for: The antibiotic furagin and its derivatives are isoform-selective human carbonic anhydrase inhibitors
Source: J Enzyme Inhib Med Chem. 2020 Apr 16;35(1):1011–20. doi: 10.1080/14756366.2020.1752201 (PMC7178874; doi:10.1080/14756366.2020.1752201)
Supplement: Supplemental Material [file IENZ_A_1752201_SM0706.pdf]

Supporting information

The antibiotic Furagin and its derivatives are isoform-selective human carbonic anhydrase inhibitors

Aleksandrs Pustenko,[1,2] Alessio Nocentini,[3,4] Paola Gratteri,[4]Alessandro Bonardi,[3,4] Igor Vozny,[1] Raivis Žalubovskis,[1,2]* Claudiu T. Supuran[3]*

[1] Latvian Institute of Organic Synthesis, 21 Aizkraukles Str., Riga LV-1006, Latvia.

[2] Institute of Technology of Organic Chemistry, Faculty of Materials Science and Applied Chemistry, Riga Technical University, 3/7 Paula Valdena Str., Riga LV-1048, Latvia.

[3] Department of NEUROFARBA, Section of Pharmaceutical and Nutraceutical Sciences, University of Florence, Polo Scientifico, Via U. Schiff 6, 50019, Sesto Fiorentino, Firenze, Italy

[4] Department of NEUROFARBA, Section of Pharmaceutical and Nutraceutical Sciences, Laboratory of Molecular Modeling Cheminformatics & QSAR, University of Florence, Polo Scientifico, Via U. Schiff 6, 50019 Sesto Fiorentino, Firenze, Italy

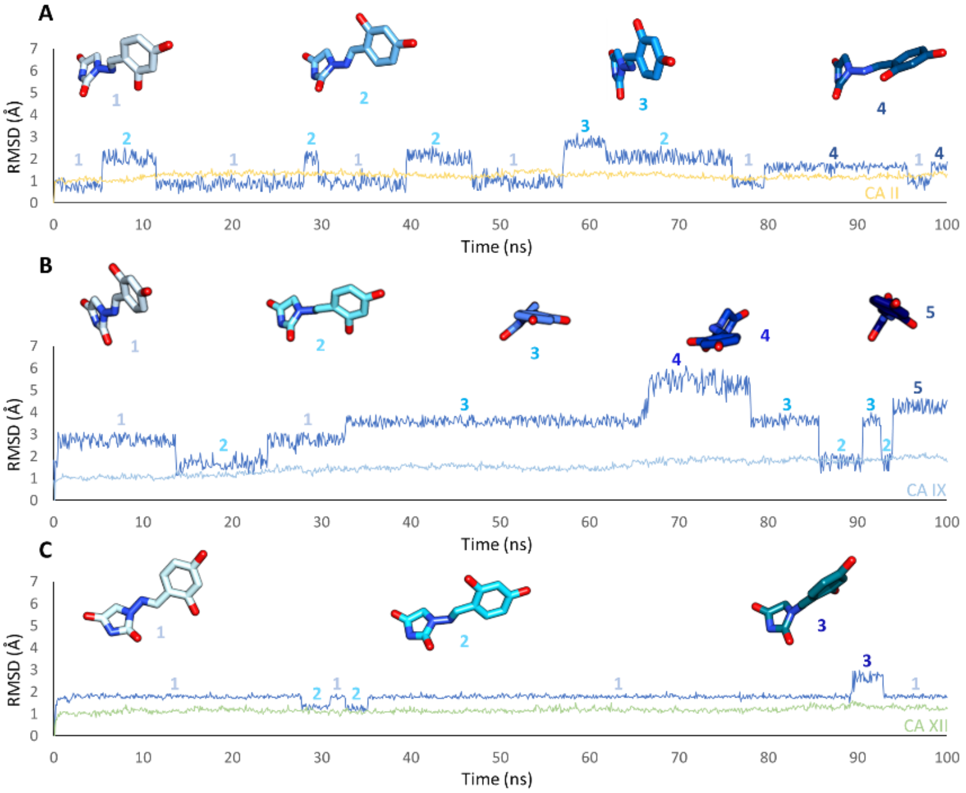

Figure S1. RMSD analysis of **12** heavy atoms and A) CA II, B) CAIX and C) CA XII backbone over the 100 ns MD simulation. The ligand color darkens over the dynamic simulation.

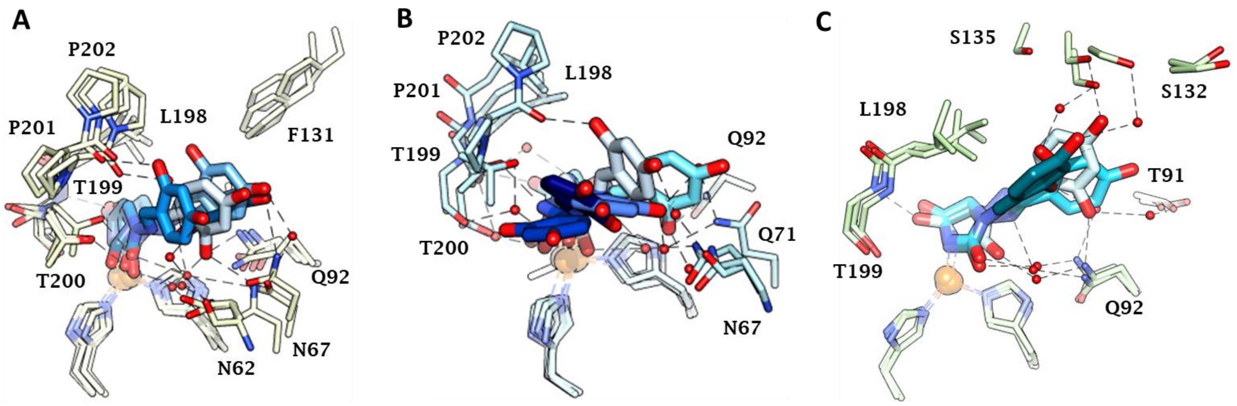

Figure S2. Dynamics evolution of the binding mode of **12** to A) CA II, B) CA IX and C) CA XII over the course of 100 ns. Water molecules are represented as red spheres. The ligand color darkens over the dynamic simulation.
